# FBXO17 promotes cell proliferation through activation of Akt in lung adenocarcinoma cells

**DOI:** 10.1186/s12931-018-0910-0

**Published:** 2018-10-25

**Authors:** Tomeka L. Suber, Ina Nikolli, Michael E. O’Brien, James Londino, Jing Zhao, Kong Chen, Rama K. Mallampalli, Yutong Zhao

**Affiliations:** 1 Department of Medicine, the Acute Lung Injury Center of Excellence, University of Pittsburgh, Pittsburgh, PA 15213 USA; 2 Department of Cell Biology, Physiology, and Bioengineering, University of Pittsburgh, Pittsburgh, PA 15213 USA; 3 Medical Specialty Service Line, Veterans Affairs Pittsburgh Healthcare System, Pittsburgh, PA 15240 USA; 4 Department of Medicine, Pulmonary, Allergy, & Critical Care Medicine, The University of Pittsburgh, UPMC Montefiore, NW 628, Pittsburgh, PA 15213 USA

**Keywords:** Akt, FBXO17, Proliferation, Lung cancer

## Abstract

**Background:**

The ubiquitin-proteasome pathway, mediated in part, by ubiquitin E3 ligases, is critical in regulating cellular processes such as cell proliferation, apoptosis, and migration. FBXO17 was recently identified as an F-box protein that targets glycogen synthase kinase-3β to the E3 ubiquitin ligase protein complex for polyubiquitination and proteasomal degradation. Here, we identified that in several lung adenocarcinoma cell lines, FBXO17 cellular protein was detected at relatively high levels, as was expression in a subset of lung cancers. Hence, we investigated the effects of *FBXO17* on cell proliferation.

**Methods:**

Single cell RNA sequencing analysis was performed on a resection of a non-small cell lung carcinoma tumor to examine *FBXO17* expression. Multiple lung cancer cell lines were immunoblotted, and The Cancer Genome Atlas was analyzed to determine if FBXO17 expression was amplified in a subset of lung cancers. A549 cells were transfected with empty vector or *FBXO17-V5* plasmid and immunoblotted for Akt pathway mediators including PDK1, ERK1/2, ribosomal protein S6, and CREB. Cell proliferation and viability were analyzed by trypan blue exclusion, BrdU incorporation and an MTS-based fluorometric assay. Studies were also performed after transfecting with *sifbxo17.* Samples were used in an RNA microarray analysis to evaluate pathways affected by reduced *FBXO17* gene expression.

**Results:**

We observed that overexpression of *FBXO17* increased A549 cell proliferation coupled with Akt activation. Ectopically expressed *FBXO17* also increased ERK1/2 kinase activation and increased phosphorylation of RPS6, a downstream target of mTOR. We also observed an increased number of cells in S-phase and increased metabolic activity of lung epithelial cells expressing FBXO17. *FBXO17* knockdown reduced Akt Ser 473 phosphorylation approaching statistical significance with no effect on Thr 308. However, ERK1/2 phosphorylation, cellular metabolic activity, and overall cell numbers were reduced. When we analyzed RNA profiles of A549 cells with reduced FBXO17 expression, we observed downregulation of several genes associated with cell proliferation and metabolism.

**Conclusions:**

These data support a role for FBXO17 abundance, when left unchecked, in regulating cell proliferation and survival through modulation of Akt and ERK kinase activation. The data raise a potential role for the F-box subunit in modulating tumorigenesis.

**Electronic supplementary material:**

The online version of this article (10.1186/s12931-018-0910-0) contains supplementary material, which is available to authorized users.

## Background

The phosphatidylinositol-4,5-bisphosphate 3-kinase (PI3K)-Akt- mammalian target of rapamycin (mTOR) pathway drives many cellular functions including cell survival, proliferation, and migration. Activation of receptor tyrosine kinases (RTKs) including EGFR, Her2/Neu, and VEGF activates and recruits PI3K to the plasma membrane [1]. Subsequent phosphorylation of phosphatidylinositol-4,5-bisphosphate (PIP_2_) generates phosphatidylinositol-3,4,5-trisphosphate (PIP_3_) as a second messenger to recruit and activate Akt through phosphorylation by PDK1. Akt then phosphorylates downstream targets such as Bad, GSK3β, and FOXO transcription factors to inhibit apoptosis and promote cell survival, migration, and proliferation [2, 3]. Akt activation occurs on serine 473, the obligatory catalytic site, and threonine 308, a tightly regulated site that is required for maximal Akt activation. Phosphatase and tensin homolog (PTEN) is well-characterized as the critical negative regulator of Akt activity through dephosphorylation of PIP_3_ to PIP_2_ [4]. A critical substrate of Akt is mTOR, a serine-threonine kinase that forms part of two complexes mTORC1 and mTORC2. While the primary targets of mTORC1 include p70S6 Kinase 1 (S6K1), RPS6, and eIF4E Binding Protein (4EBP) to promote protein translation, mTORC2 facilitates cell growth through phosphorylation of downstream targets such as several members of the AGC (PKA/PKG/PKC) family of kinases in addition to Akt [5, 6].

Mutations, amplifications, and hyperactivity of the PI3K-Akt-mTOR pathway can lead to aberrant cell growth and tumorigenesis [7]. Activating mutations in *PIK3CA* encoding PI3K occur in a large number of lung cancers [8, 9]. Mutations in *PTEN* are among the highest frequency mutations in all cancers [10–12]. A large number of mTOR mutations have been identified in several malignancies, some of which confer constitutive activation to the kinase [13]. A majority of lung cancers have high levels of mTOR pathway activation, and phosphorylation of S6K is associated with metastasis and poor survival in adenocarcinoma [14]. Developing therapies with more specific targeting of the mTOR pathway based on molecular profiling of tumors is an intense area of research. In non-small cell lung cancers (NSCLC), mutations in *NF1*, *MET*, and *ERBB2* account for up to 13% of tumors analyzed by molecular profiling, and elevation in MAPK and PI3K activity was observed in a large proportion of cases [15].

The cellular concentrations of key effectors that drive malignant phenotypes within cellular signaling pathways such as the PI3K/Akt/mTOR signaling cascade are partly controlled at the level of protein stability [16–18]. The ubiquitin-proteasome pathway is the primary mechanism for degradation of cellular proteins in eukaryotic cells [11, 19]. Regulation of protein stability is critical for cellular homeostasis, and disruption can lead to aberrant cell proliferation. The final step in targeting proteins for proteasomal degradation is transfer of polyubiquitin chains to the targeted substrates by an E3 ubiquitin ligase. The Skp-Cullin-F-box (SCF) family is the largest family of E3 ubiquitin ligases, comprised of multiple subunits that execute ubiquitination of targets through a substrate recognition module, termed an F-box protein. There are ~ 70 F-box proteins, many of which have not been characterized [20]. Proteins undergo post-translational modifications, usually phosphorylation, to generate a “degron” that is recognized by the E3 ubiquitin ligase complex [21, 22]. Dysregulation of several F-box proteins have been linked to cancer. For example, Fbxw7 targets mTOR, c-Myc, c-Jun, cyclin E, and several other proteins implicated in oncogenesis, thus functioning as a tumor suppressor [23]. Mutations in *FBXW7* are highly represented in bile duct cancers and T-cell acute leukemia, and a large proportion are located in the domain required for substrate recognition [24]. Bcl-6, a proto-oncogene overexpressed in diffuse large B-cell lymphoma (DLBCL), is targeted by FBXO11 for polyubiquitination and degradation [25]. In a number of DLBCL lines FBXO11 was found to be mutated or deleted, and restoration of FBXO11 expression in DLBCL-derived tumor cells in immunodeficient mice induced apoptosis and suppressed tumor growth.

A poorly studied F-box protein, FBXO17, was recently found to be robustly expressed in murine and human lung alveolar epithelial cells [26]. We previously characterized FBXO17 as a negative regulator of glycogen synthase kinase-3β (GSK3β) through polyubiquitination and targeting of the kinase to the proteasome for degradation [26]. Because Akt phosphorylates and negatively regulates GSK3β, a potentially important association that might impact cell growth and survival, we tested the hypothesis that FBXO17, highly expressed in lung carcinoma, regulates cell proliferation through Akt activation. We found that FBXO17 promotes cell proliferation with associated PI3K/Akt/mTOR and ERK pathway activation.

## Methods

### Antibodies and reagents

FBXO17 antibody was obtained from Origene (cat# TA331636, Rockville, MD, dilution 1:1000). HRP-conjugated anti-mouse IgG and anti-rabbit IgG were obtained from BioRad (Hercules, CA, dilution 1:2000). Antibodies against Akt (cat# 2920S, dilution 1:1000), p-Akt-Ser473 (cat# 4060S, dilution 1:1000), p-Akt-Thr308 (cat# 13038S, dilution 1:1000), GSK3β (cat# #9315, dilution 1:1000), p- GSK3β (Ser9) (cat# 9336, dilution 1:1000), CREB (cat# #9197, dilution 1:1000), p-PDK1 (cat# 3438, dilution 1:1000), ERK1/2 (cat# 9101S, dilution 1:1000), p-ERK1/2 (cat# 4696S, dilution 1:1000) p-mTOR (cat# 5536 T, dilution 1:1000), and mTOR (cat# 2983 T, dilution 1:1000) were obtained from Cell Signaling (Beverly, MA). PDK1 (cat# ab52893, dilution 1:1000) and p-CREB (cat# ab32096, dilution 1:1000) antibodies were purchased from Abcam (cat# ab52893, Cambridge, MA). RPS6 antibody (cat# sc-74459, dilution 1:1000) was purchased from Santa Cruz. β actin antibody was obtained from Sigma (St. Louis, MO, USA, dilution 1:30000). Mouse anti-V5 antibody (ref# 46–0705, dilution 1:5000) and midiprep kits were purchased from Invitrogen (Grand Island, NY). BrdU flow cytometry assay was purchased from BD Biosciences (San Jose, CA). Lipofectamine 3000 was purchased from Thermo Fisher (Waltham, MA) and Gene Mute was purchased from SignaGen (Rockville, MD). Trypan blue stain (0.4%) was purchased from GIBCO (Life Technologies, Grand Island, NY).

### Cell culture and transfection

Human A549 cells were obtained from ATCC (Manassas, VA, USA). RPMI medium was purchased from GIBCO (Life Technologies, Grand Island, NY). Cells were supplemented with 10% fetal bovine serum (FBS) from Gemini (Sacramento, CA, USA). Cloning of FBXO17 was described previously [26]. Cells were seeded at a density of 5 × 10^5^ in a 6-well plate and transfected with 2 μg *FBXO17*-V5 plasmid or empty vector with Lipofectamine 3000 according to the manufacturer’s protocol. For gene silencing, scrambled and silencing siRNAs were purchased from Integrated DNA Technologies (Coralville, IA). Scrambled control siRNA (cat# 51–01–19-09) and two siRNAs targeting *FBXO17* (design ID# hs.Ri.FBXO17.13.1 and hs.Ri.FBXO17.13.2) were used in dual transfections for knockdown for in vitro and immunoblotting experiments. Gene Mute was used for transfection according to manufacturer’s protocol. Lysates were prepared 72 and 96 h post-transfection.

### Immunoblotting

Cell lysates in 150 μl of lysis buffer (20 mM Tris-HCl (pH 7.4), 150 mM NaCl, 2 mM EGTA, 5 mM β-glycerophosphate, 1 mM MgCl2, 1% Triton X-100, 1 mM sodium orthovanadate, 10 μg/ml protease inhibitors, 1 μg/ml aprotinin, 1 μg/ml leupeptin, and 1 μg/ml pepstatin) were sonicated on ice for 12 s and centrifuged at 10000×g for 10 min at 4 °C in a microcentrifuge. After overexpression, lysates were prepared at 48 h post-transfection. For knockdown, lysates were prepared 72 h post-transfection. For immunoblotting, equal amounts of supernatant (15 μg protein) were subjected to 10% SDS-PAGE gels, transferred to nitrocellulose membranes, blocked with 5% (*w*/*v*) bovine serum albumin in TBST (25 mM Tris-HCl, pH 7.4, 137 mM NaCl, and 0.1% Tween 20) for 1 h, and incubated with primary antibodies in 5% (w/v) BSA in TBST for 1–2 h. The membranes were washed at least three times with TBST at 10-min intervals followed by a 1 h incubation with mouse, rabbit, or goat horseradish peroxidase-conjugated secondary antibody (1:2000). The membranes were developed with an enhanced chemiluminescence detection system according to manufacturer’s instructions.

### Cell cycle and viability assays

For cell counting, 5 × 10^5^ cells were seeded per well in a 6-well plate and transfected with *FBXO17-V5* or empty plasmid. After 48 h, cells were trypsinized and diluted 1:1 in trypan blue in a 96-well plate for cell counting. Cell viability was determined by trypan exclusion and 20 fields were counted per condition using an automated cell counter (TC20, BioRad). For *FBXO17* silencing, scrambled or *siFBXO17* siRNAs were transfected after seeding 2 × 10^5^ cells/well. After 96 h, cells were counted as described with viability determined by trypan blue exclusion. For overexpression, A549 cells were plated at 5 × 10^4^ cells per well in triplicate per condition in a 96-well plate 24 h after transfection of FBXO17-V5 plasmid in 100 uL. At 24, 48, and 72 h after seeding, the MTS ([3-(4,5-dimethylthiazol-2-yl)-5-(3-carboxymethoxyphenyl)-2-(4-sulfophenyl)-2H-tetrazolium) assay with conversion to a soluble formazan product was performed according to manufacturer’s protocols (Promega CellTiter 96® AQueous One Solution Cell Proliferation Assay). After adding 20 uL of reagent, the plate was read at 490 nm for absorbance per protocol. For knockdown experiments, 5 × 10^4^ cells/well were seeded 48 h post transfection. The assays were performed at 24, 48, and 72 h after seeding. For cell cycle assays, A549 cells were transfected with empty vector or *FBXO17*-V5 plasmid. After 24 h, cells were cultured in serum-free medium overnight for 16 h. Cells were then pulsed with BrdU for 45 min and returned to culture in 10% serum for 6 h. Cells were fixed, permeabilized, and treated with DNAase prior to staining with anti-BrdU-APC antibody according to the manufacturer’s protocol. 7-AAD was added and cells were analyzed by flow cytometry. Data analysis was performed using FlowJo Software (version 10.4.1). Similar analysis was performed for knockdown at 72 h post-transfection.

### Single cell RNA sequencing

To determine the cell specificity of FBXO17 expression, we analyzed single-cell gene expression data from a fresh surgical resection of a non-small cell lung carcinoma tumor generated by 10x Genomics, Inc. (https://support.10xgenomics.com/single-cell-vdj/datasets/2.1.0/vdj_v1_nsclc_5gex). Two-dimensional T-distributed stochastic neighbor embedding (t-SNE) plots were generated based on global gene expression relationships among 7,802 total cells. FBXO17 and three marker genes, including PTPRC (leukocytes), SCGB1A1 (club cells), and EPCAM (lung epithelial cells) were displayed with level of expression indicated by intensity of red.

### RNA microarray analysis and gene enrichment analysis

A549 cells were transfected with control scrambled siRNAs or *siFBXO17* (Integrated DNA Technologies). After 72 h cells were harvested and RNA isolated using the Invitrogen RNEasy Plus kit (3 independent control and 3 *siFBXO17* samples). After confirming knockdown of *FBXO17* expression by quantitative real time PCR, RNA was analyzed using the Clariom S Assay (Affymetrix and Thermo Fisher Scientific, cat# 902926). Preparation of cDNA was performed using the WT Plus reagent kit (Thermo Fisher) and according to manufacturer instructions. Reverse transcription was performed using 100 ng total RNA and dNTP-T7 random primers. Second strand synthesis and in vitro transcription created amplified cRNA. cDNA from a second reverse transcription reaction was fragmented and end labeled with biotin for hybridization to the Clariom S human array. Following overnight (16 h) hybridization at 45 °C with rotational mixing at 60 rpm, arrays were processed on a GeneChip® 450 Fluidics Station using manufacturer specified protocols. To remove unbound sample, arrays were first washed with non-stringent wash buffer A. The GeneChips® were then stained for 10 min in Stain Cocktail 1. Buffer A was again used to wash off excess stain. Signal amplification was achieved by 10 min incubation with Stain Cocktail 2 followed by a second 10 min incubation with Stain Cocktail 1. The chip was washed with high stringency Buffer B and filled with Array Holding Buffer before being removed from the fluidics station and scanned using the GeneArray® 3000 scanner. First level image analysis was performed using Affymetrix Expression Console. Normalization was via robust multiarray average (RMA).

### Gene enrichment analysis

For pathways analysis, average log 2-fold change expression was determined in each of three technical replicates and an average fold change compared to *Fbxo17* knockdown was calculated. Differentially expressed genes (DEGs) used in pathway and Gene Ontology (GO) analysis were determined using Transcriptome Analysis Console version 4.0.1 (Applied Biosystems, Foster City, CA). Filters were used to select genes with absolute fold change (FC) < − 1.5 and > 1.5 and a *P*-value ≤0.05. Gene Ontology (GO) enrichment analysis was performed using ToppGene (http://toppgene.cchmc.org/) [27]. Correction for multiple comparisons was performed using the Benjamini–Hochberg procedure and significance determined using a false discovery rate (FDR) *P*-value cut-off < 0.05. Activation or inhibition of gene pathways was evaluated with Ingenuity Pathway Analysis (IPA, Qiagen), which compares results to experimentally determined gene expression changes reported in the literature.

### Statistical analysis

Descriptive statistics were reported with mean ± standard deviation (SD) or standard error (SE) indicated. Our sample sizes of each experimental group were all less than 10 that limited ability to study a normal sample distribution. Thus, we employed appropriate non-parametric methods such as a Mann-Whitney U test and a Kruskal-Wallis equality-of-populations rank test to compare multiple groups within and between experiments. Also, a Wilcoxon-type test for trend was used where appropriate to check trends in data significance. Using these methods provided conservative analysis to determine statistical significance. Densitometry was performed using Image J software Version 2.0.0-rc-67/1.52d [28]. All analyses were performed using one-way ANOVA or paired t-tests using GraphPad Prism version 7.0 for Windows and Mac OS, GraphPad Software, La Jolla California USA, www.graphpad.com.

## Results

### FBXO17 is highly expressed in some lung cancer cell lines

**Fig. 1:**
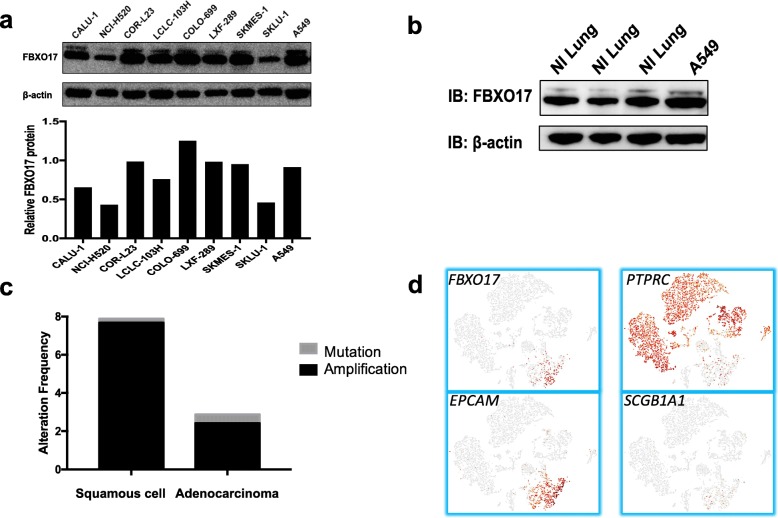
FBXO17 is highly expressed in a subset of lung cancer cell lines and primary lung bronchogenic tumors. **a** Cell lysates of lung cancer cell lines were immunoblotted for FBXO17 protein expression with β-actin as a loading control. **b** Normal lung tissue and A549 lysates were immunoblotted for FBXO17 protein with β-actin as a loading control. **c** FBXO17 gene expression amplification was observed in a subset of lung squamous cell carcinoma and adenocarcinoma cases. The results presented here are in whole or part based upon data generated from the Cancer Genome Atlas Network: http://cancergenome.nih.gov/ (*n* = 1144 samples). **d** RNA-sequencing of resected non-small cell lung cancer tumor is displayed in a t-distributed stochastic neighbor embedding (tSNE) plot showing RNA expression of cell-specific markers including epithelial adhesion molecule (EPCAM, epithelial cells), protein tyrosine phosphatase receptor type C (PTPRC, leukocytes), and secretoglobin family 1A member 1 (SCGB1A1, club cells)

We first examined if FBXO17 was overexpressed or altered in lung cancer cell lines (Fig. 1a). Relative to several cell lines examined, COR-L23, COLO-699, and A549 expression of FBXO17 protein was detected most abundantly. A549 cells express 40% more FBXO17 protein relative to normal lung tissue (Fig. 1b). We also interrogated the cBio Cancer Genomics Portal and found that FBXO17 expression was increased in 5% of all lung cancer samples in the database (*n* = 1144) [15, 29, 30]. Amplification of gene expression was disproportionately high in squamous cell lung cancers at 7.6% of cases compared to 2.9% of non-small cell lung cancer (NSCLC) cases (Fig. 1c). Single cell RNA-sequencing data was obtained from resected squamous cell carcinoma tumor from 10x Genomics, Inc. (Fig. 1d**)**. As shown in the t-distributed stochastic neighbor embedding (tSNE) plot, FBXO17 co-localizes with the epithelial cell marker EPCAM and is highly expressed in epithelial cells while there was no expression in leukocytes (PTPRC expression) or club cells (SGCB1A1). These data suggest that FBXO17 may be dysregulated in a subset of bronchogenic carcinomas.

### FBXO17 increases activation of the PI3K-Akt-mTOR signaling pathway in A549 cells

**Fig. 2:**
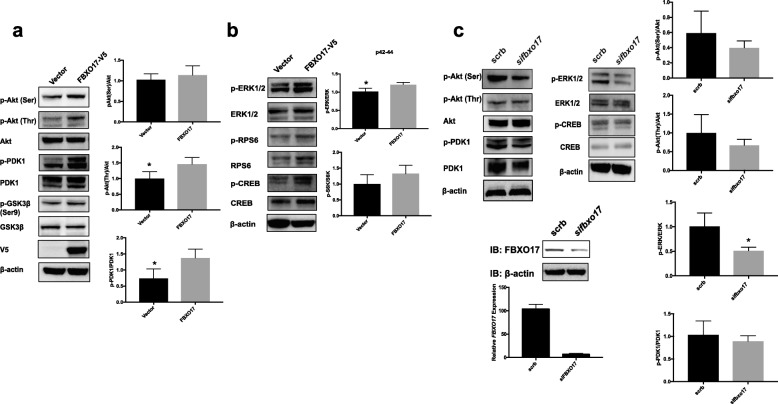
FBXO17 increases Akt activation through site-specific phosphorylation. **a** A549 cells were transfected with *FBXO17*-V5 plasmid or empty vector. Lysates were prepared at 48 h after transfection and immunoblotted for indicated kinases. **b** Analysis of phosphorylation bands for kinases was quantitated using densitometry and results graphed as shown. Analysis of protein levels and relative phosphorylation were quantitated using densitometry with β-actin as a loading control and results were graphed as shown. Data is representative of three independent experiments, *p < 0.05. **c** A549 cells were transfected with scrambled siRNA and *siFBXO17*. Lysates were prepared 72 h after transfection and immunoblotted. Analysis of protein levels and relative phosphorylation were quantitated using densitometry with β-actin as a loading control and results were graphed as shown. Quantitative RT-PCR and immunoblotting were performed to confirm reduced *FBXO17* expression after knockdown (below). Data is shown on effect of *FBXO17* siRNA on steady-state *FBXO17* mRNA levels. Data is representative of three independent experiments, *p < 0.05

Given our prior studies supporting a role for FBXO17 in targeting GSK3β for proteasomal degradation in lung epithelial cells, we examined whether FBXO17 modulates the activity of Akt, a key negative regulator of GSK3β. When *FBXO17* was ectopically expressed in A549 cells, phosphorylation of threonine 308 (Thr 308), and to a lesser degree serine 473 (Ser 473) were increased relative to total Akt levels (Fig. 2a). Inhibitory Ser 9 phosphorylation of GSK3β was unchanged with modest reduction in total protein levels, consistent with prior published data [26]. In addition, phosphorylation of 3-phosphoinositide dependent protein kinase 1 (PDK1), extracellular signal-regulated kinase-1/2 (ERK1/2), p-CREB, and RPS6 were increased after ectopic *FBXO17* expression (Fig. 2a, b). With knockdown, there was a trend for a reduction of p-Akt (Ser473) phosphorylation while p-Akt (Thr308) was not significantly decreased. (Fig. 2c). We observed significant reductions in p-ERK1/2 with *FBXO17* knockdown while PDK1 phosphorylation also decreased but did not reach significance after densitometric analysis of bands visualized on the immunoblots. The data show that robust expression of FBXO17 is associated with increased Akt, PDK1 and ERK1/2 activation, with more modest effects upon *FBXO17* cellular depletion.

### FBXO17 increases A549 cell proliferation

**Fig. 3:**
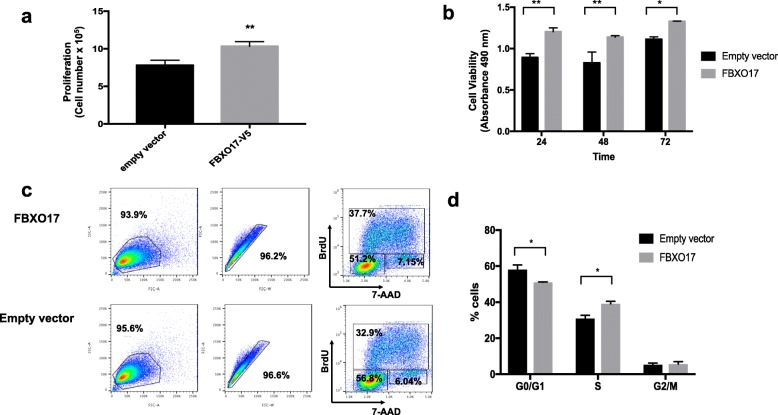
Ectopically expressed *FBXO17* increases cell proliferation and cell viability. **a** A549 cells were transfected with *FBXO17-V5* plasmid or empty vector. Live cells were counted 48 h after transfection using trypan blue exclusion staining. Data is representative of three independent experiments, **p < 0.001. **b** A549 cells were transfected with *FBXO17*-V5 plasmid or empty vector. After 24 h cells were plated at 5 × 10^4^/well in a 96-well plate. MTS was added at 24, 48, and 72 h. After 30 min, samples were analyzed by spectrophotometry at a wavelength of 490 nm. Data is representative of three independent experiments, *p < 0.01, **p < 0.001. **c** A549 cells were transfected with *FBXO17-V5* or empty vector and cultured in complete medium for 24 h. Complete medium was replaced with serum-free medium for 16 h. Cells were then pulsed with BrdU in completed medium for 45 min. After removal of BrdU, cells were cultured in complete medium for an additional 6 h. Cells were processed and stained with anti-BrdU and 7-AAD and analyzed by flow cytometry. Data in (**c**) is graphically represented for each phase and shown in (**d**). Data is representative of three independent experiments, *p < 0.01

**Fig. 4:**
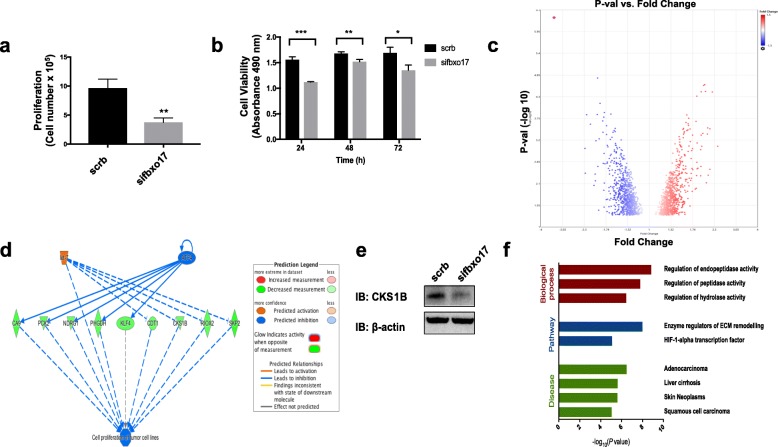
*FBXO17* depletion reduces cell proliferation and dysregulates the PI3K/Akt/mTOR pathway. **a** A549 cells were transfected with scrambled siRNA or si*FBXO17*. Live cells were counted 96 h after transfection using trypan blue exclusion staining. Data shown is representative of three independent experiments, **p < 0.001. **b** A549 cells were transfected with scrambled siRNA or empty vector. After 48 h cells were plated at 5 × 10^4^/well in a 96-well plate. The MTS reagent was added at 24, 48, and 72 h after seeding. Following 30 min of substrate addition samples were analyzed by spectrophotometry in triplicate. Data shown is representative of three independent experiments, *p < 0.05, **p < 0.01, ***p < 0.001. **c** After 72 h post transfection of scrambled siRNAs or *siFBXO17*, A549 cells were harvested in triplicates and RNA was isolated for analysis using the Clariom S RNA microarray. Using cutoffs of +/− 1.5-fold change a volcano plot was created representing 212 genes (Transcriptome Analysis Console Software, version 4.0.1). **d** Pathway analysis of Clariom S transcriptome profiling using ToppGene revealed decreased expression of nine genes involved in cell proliferation and metabolism of tumor cell lines (consistency score 2.667). **e** Immunoblot of CKS1B is shown in control (scrb) and *sifbxo17* cell lysates. **f** ToppGene was used to analyze the gene sets and determine enrichment of biological pathways and diseases

To determine if FBXO17 influenced cell survival and growth, *FBXO17*-V5 plasmid was overexpressed in A549 cells. At 48 h post-transfection cell numbers significantly increased with *FBXO17* overexpression but not with empty vector (Fig. 3a). In a metabolic MTS colorimetric assay, cell viability was also modestly enhanced at 24, 48, and 72 h post transfection (Fig. 3b). After 16 h of serum deprivation, cells were pulsed with BrdU and stimulated with 10% serum prior to staining and flow cytometric analysis. A higher fraction of cells was found to be in S phase with *FBXO17* overexpression compared to empty vector (Fig. 3c, d). When *FBXO17* expression was silenced, cell proliferation was significantly reduced (Fig. 4a, b). These results support a role for a high level of FBXO17 protein mass in modulating cell proliferative behavior, and depletion of *FBXO17* gene expression substantially impairs cell growth and survival.

### Reduced expression of FBXO17 enriches for squamous and adenocarcinoma-associated genes

When we examined pathways affected by knockdown of *FBXO17* using RNA microarray analysis, we identified enrichment of genes affected which included endopeptidase proteins and pathways, hypoxia-inducible factor 1-alpha (HIF1α) pathway genes, and both squamous cell cancer and adenocarcinoma-associated genes (Fig. 4c-f). Gene expression analysis revealed 212 genes that were differentially expressed in A549 cells that underwent *FBXO17* knockdown (100 downregulated and 112 upregulated) (Fig. 4c). Pathway analysis revealed decreased expression of nine genes involved in cell proliferation and metabolism of tumor cell lines, indicating a role for upstream inhibition of the transcriptional regulator ATF4 and upstream activation of let-7 in our model of *FBXO17* knockdown (Fig. 4d). We confirmed CKS1B reduction by immunoblotting (Fig. 4e). Gene enrichment analysis of these differentially expressed genes revealed that regulation of endopeptidase and peptidase activity (FDR *p*-value 5.84 e-03) were the most significant biological processes involved in the experimental model of *FBXO17* knockdown. Enzymatic regulators of extracellular matrix remodeling were the most significant pathway affected (FDR *P*-value 2.87 e-04). Adenocarcinoma was the disease category most significantly associated with DEGs in our model (FDR P-value 8.97 e-03), (Fig. 4f). Additional genes and pathways that are differentially expressed are included in Additional files 1 and 2. In summary, several genes shown to be differentially expressed when FBXO17 is depleted are implicated in metabolic and cell proliferative functions that may provide further insight into the role of this poorly studied F-box protein in cancer biology.

## Discussion

The role of FBXO17 in the regulation of cell growth and survival to date is unknown. FBXO17 mediates degradation of GSK3β, a purported inhibitor of cell proliferative activity, that in turn is inhibited by Akt [31]. These data together with findings that Akt participates in the pathobiology of tumorigenesis led us to investigate if FBXO17 is differentially expressed in lung carcinoma. Indeed, FBXO17 was detected at high levels in many lung carcinoma cell lines and its transcript is highly expressed in squamous cell lung cancer. When overexpressed in A549 cells, *FBXO17* increased cellular proliferative activity by ~ 25% coupled with increased survival of cells. We also demonstrate here that Akt phosphorylation on Thr 308, and to a lesser extent Ser 473, is enhanced by ectopic *FBXO17* expression. Interestingly, Ser 473 phosphorylation was reduced more than Thr 308 with *FBXO17* knockdown. Our studies support a correlation between higher levels of FBXO17 and Thr 308 activity. A number of genes implicated in key pathways and biological processes that are linked to tumorigenesis were modulated after *FBXO17* silencing in cells. These results suggest that FBXO17 is sufficient to elicit pro-survival effects in some cell lines. Depletion of *FBXO17* gene expression substantially decreases replicative behavior of these cells.

Proliferation of A549 cells was clearly enhanced by ectopic *FBXO17* expression. Akt activation by PDK1 occurs in a two-step process. Serine 473 phosphorylation is obligatory for activation; however, maximal activation requires subsequent phosphorylation of threonine 308 [2, 6, 32]. Phosphorylation of both sites is tightly regulated and influences downstream signaling and substrate selection. It has been proposed that the balance of Ser 473 and Thr 308 phosphorylation is integrated to regulate downstream signals [2]. Akt activity is fine-tuned by phosphorylation on additional sites by other upstream kinases [33]. In our overexpression studies, ectopically expressed FBXO17 seemed to clearly upregulate Thr 308 phosphorylation to a greater extent than serine 473. The converse was true with knockdown where Ser 473 phosphorylation was reduced relative to controls, trending toward significance. Little change in Thr 308 phosphorylation was observed. Threonine 308 has been shown to be more closely associated with Akt activity in non-small cell lung cancer samples than serine 473 [34]. With high glucose culture medium, only Ser 473 seemed to be affected by FBXO17 expression, and depletion of *FBXO17* did not have significant effects on proliferation (unpublished data). These observations suggest that a delicate balance between serine and threonine phosphorylation can be perturbed by FBXO17 with downstream effects on cell survival and proliferation, and glucose metabolism influences these responses. Phosphorylation of PDK1 as well as mediators further downstream in the PI3K-Akt-mTOR pathway were also activated. Cyclic AMP responsive element binding protein (CREB) activation has been shown to be enhanced by Akt and inhibited by GSK3β [35–37]. Akt-dependent Ser 133 phosphorylation promotes recruitment of CREB binding protein (CBP) to activate gene expression to promote cell survival [37]. Increased FBXO17 levels were associated with increased CREB phosphorylation at Ser 133, a site known to enhance CREB activation. Using transcriptome profiling and pathway analysis after *FBXO17* knockdown, CKS1B and SKP2 were notable genes that were differentially expressed and important in regulating cell cycle progression through Skp2-mediated degradation of the tumor suppressor p27^Kip^ [38, 39] Other genes were affected including genes important in metabolism such as PHGDH, RIOX2, and KLF4 that have been shown to have pleotropic roles in chemotherapeutic resistance in different cancers [40–42]. It will be interesting to explore how dysregulation of Akt-dependent pathways by FBXO17 can perturb additional downstream signaling in pathways critical for regulating cell survival and apoptosis.

he magnitude of some of the biological and biochemical processes observed here at times were modest, but can be partly explained by the observation that A549 cells that were used are derived from adenocarcinoma cells with high baseline FBXO17 protein. Thus, overexpression of this F-box protein may have more limited effects biologically given saturation of FBXO17 concentrations in cells that act as a molecular input to pro-survival pathways. ERK1/2 phosphorylation was significantly increased with FBXO17 overexpression and reduced with FBXO17 knockdown, a more pronounced effect while Thr 308 Akt activation is unchanged with FBXO17 knockdown. These data suggest there are alternate mechanisms independent of maximal Akt activation that promote cell proliferation, and future studies will explore the role of the Ras/Raf/ERK kinase pathway in FBXO17-mediated cell proliferation. Prior studies have described ERK-dependent signaling promoting RPS6 phosphorylation [43]. Nevertheless, sustained high cellular concentrations with even modest activation of these cell signaling pathways may translate into a more aggressive cell phenotype with regard to neoplastic behavior. Hence, in future studies the kinetics and magnitude of sustained or conditional *FBXO17* expression in a clonal cell line that is devoid of endogenous F-box protein or in vivo system may be of interest.

With the growth of rich databases of genomic data available for lung cancer, molecular details regarding mutations, deletions, and amplification of genes can be identified to target therapy. In a recent study by Chen and colleagues, nine subtypes of NSCLC were characterized, and for adenocarcinoma three subtypes showed significant increases in p38 and mTOR pathway activation [44]. Several cancer cell lines have been characterized by having mutations in the mTOR pathway [45]. A high proportion of samples in the original study that formed the basis of The Cancer Genome Atlas showed increased MAPK and mTOR pathway activation, but gene mutations identified could only explain a fraction of these cases [15]. The study illustrates that other pathways leading to activation of mTOR have yet to be identified. Interestingly, a recent study showed that overexpression of a de-ubiquitin enzyme termed ubiquitin C-terminal hydrolase L1 (UCH-L1) promoted Akt activation in MCF-7 breast cancer cells without altering proliferation and enhanced cellular invasion [46]. *FBXO17* expression appears to be advantageous for tumor cell survival, and we propose that upregulation of Akt activation by this F-box protein is a potential mechanism for increased mTOR activation in cancer. The results here may be clinically relevant given the interest in personalized, targeted therapies that are currently in use in lung cancer such as use of tyrosine kinase inhibitors for patients with KRAS mutations observed in many NSCLC patients [1, 8, 47, 48].

One limitation of our study is that while we see trends for reduced Akt Ser 473 phosphorylation, this did not reach statistical significance. Akt Thr 308 is consistently unchanged in our experiments with *FBXO17* knockdown. CREB is also not significantly reduced with *FBXO17* knockdown in line with its role as a downstream target of Akt, suggesting that overall Akt activity remains high, albeit somewhat reduced, with *FBXO17* silencing. In our functional assays however, *FBXO17* silencing is associated with a significant reduction in cell proliferation, suggesting Akt-independent functional consequences through dysregulated FBXO17 expression. Interestingly we do not observe significant differences in cell cycle progression using serum starvation and BrdU assays (data not shown) suggesting that more robust cellular depletion of *FBXO17* may be necessary. We also observed that ERK1/2 was modulated through *FBXO17* ectopic expression or depletion, suggesting that other pathways partake in SCF-FBXO17 signaling. In addition, phosphatases PP2A, known to target Thr 308, and PHLPP, which dephosphorylates Ser 473, may be differentially regulated downstream of FBXO17 to explain these results [49]. More complete gene depletion by CRISPR/Cas9 may yield more robust reduction in Akt phosphorylation with *FBXO17* gene silencing. High FBXO17 expression, however, drives cell proliferation and may be relevant in the context of lung cancer and aberrant cell proliferation.

## Conclusions

Our studies demonstrate a role for FBXO17 in cell proliferation in lung epithelial cells with some specificity for Ser 473 phosphorylation of Akt. An association was found between FBXO17 expression and lung cancer using the large Cancer Genome Atlas cohort. In addition, downstream targets of Akt including CREB and RPS6 were found to be increased, and ERK1/2 was activated with overexpression of *FBXO17,* an effect reversed with *FBXO17* knockdown, suggesting that these proliferative effects are, in part, Akt-independent. It has also been shown that while RPS6 is phosphorylated by mTOR, Ras/ERK signaling also leads to RPS6 phosphorylation [43, 50]. A subset of lung cancers highly express *FBXO17* mRNA transcript and protein, and our data show that FBXO17 promotes robust cell proliferation and cell cycle progression that is potentially relevant in non-small cell lung cancer. These data suggest that while FBXO17 has marked effects on Ser 473 Akt phosphorylation, the F-box protein likely also regulates the Ras/ERK signaling pathway independent of Akt, raising intriguing questions for complementary pathway activation.

It is unclear if the polyubiquitination function of FBXO17 is required for activation of Akt. F-box proteins are capable of recruiting substrates and regulating pathways without proteasomal degradation. For example, FBXO17 itself has been shown to recruit protein phosphatase 2A (PP2A) to interferon regulatory factor 3 (IRF3) to facilitate dephosphorylation of IRF3, thus inhibiting dimerization and translocation to the nucleus [51]. As a result, type I interferon signaling is reduced. Thus, in future studies it is of interest to elucidate if FBXO17 mediates Akt activation through such mechanisms that are not post-translational, or via polyubiquitination and degradation of molecular inputs that inhibit Akt actions. Future studies will also be needed to identify additional substrates of FBXO17 that have potential roles in tumorigenesis and determine if these effects are mediated by polyubiquitination or if FBXO17 serves as a molecular scaffold for other proteins to regulate the PI3K-Akt-mTOR pathway.

## Additional files

Additional file 1: RNA microarray analysis using Transcriptome Analysis Console version 4.0.1. (DOCX 60 kb)

Additional file 2: Gene ontology enrichment analysis using ToppGene. (DOCX 21 kb)

## Abbreviations

4EBP: eIF4E binding protein
Akt: Protein kinase B (PKB)
BRDU: Bromodeoxyuridine
CKS1B: CDC28 protein kinase regulatory subunit 1B
CREB: Cyclic AMP response element binding protein
DEGs: Differentially expressed genes
DLBCL: Diffuse large B-cell lymphoma
EGFR: Epidermal growth factor receptor
ERK1/2: Extracellular signal-regulated kinase 1/2
FBXO17: F-box only protein 17
GSK3β: Glycogen synthase kinase 3 beta
IRF3: Interferon regulatory factor 3
KLF4: Kruppel-like factor 4
MAPK: Mitogen-activated protein kinase
mTOR: Mechanistic target of rapamycin
mTORC: Mammalian target of rapamycin complex 1 or 2
MTS: (3-(4,5-dimethylthiazol-2-yl)-5-(3-carboxymethoxyphenyl)-2-(4-sulfophenyl)-2H-tetrazolium)
NSCLC: Non-small cell lung cancer
PDK1: 3-phosphoinositide-dependent protein kinase-1
PHGDH: Phosphoglycerate dehydrogenase
PHLPP: PH domain and leucine rich repeat protein phosphatase
PI3K: Phosphoinositide 3-kinase
PIP_2_: Phosphatidylinositol-4,5-bisphosphate
PIP_3_: Phosphatidylinositol-3,4,5-trisphosphate
PP2A: Protein phosphatase 2A
PTEN: Phosphatase and tensin homolog
RIOX2: Ribosomal oxygenase 2
RPS6: Ribosomal protein S6
RTK: Receptor tyrosine kinase
SCF: Skp-Cullin-F-box
Ser: Serine
Thr: Threonine
tSNE: T-distributed stochastic neighbor embedding
VEGF: Vascular endothelial growth factor
