# Supplementary material for: FBXO17 promotes cell proliferation through activation of Akt in lung adenocarcinoma cells
Source: Respir Res. 2018 Oct 25;19:206. doi: 10.1186/s12931-018-0910-0 (PMC6203195; doi:10.1186/s12931-018-0910-0)
Supplement: Supplementary file 1 — RNA microarray analysis using Transcriptome Analysis Console version 4.0.1. (DOCX 60 kb) [file 12931_2018_910_MOESM1_ESM.docx]

TABLE S1

|  |  |  |  |  |  |  |  |  |
| --- | --- | --- | --- | --- | --- | --- | --- | --- |
| Symbol | EntrezGene ID | Description | Imported ID | Fold change | P-Value | 1 Avg (log2) | 2 Avg (log2) | Rank |
| KRT222 | 125113 | keratin 222 | KRT222 | 2.4 | 0.0009 | 5.94 | 7.21 | 2 |
| GJB1 | 2705 | gap junction protein, beta 1, 32kDa | GJB1 | 2.24 | 3.80E-05 | 8.62 | 9.78 | 5 |
| ADH1C | 126 | alcohol dehydrogenase 1C (class I), gamma polypeptide | ADH1C | 2.17 | 0.0034 | 6.86 | 7.98 | 7 |
| TPPP3 | 51673 | tubulin polymerization-promoting protein family member 3 | TPPP3 | 2.17 | 0.0003 | 6.55 | 7.67 | 7 |
| OR4C6 | 219432 | olfactory receptor, family 4, subfamily C, member 6 | OR4C6 | 2.13 | 0.0049 | 3.67 | 4.76 | 9 |
| CST4 | 1472 | cystatin S | CST4 | 2.11 | 0.0003 | 11.38 | 12.46 | 11 |
| CST2 | 1470 | cystatin SA | CST2 | 2.1 | 0.0004 | 9.89 | 10.96 | 12 |
| LARGE | 9215 | like-glycosyltransferase | LARGE | 2.07 | 0.0075 | 3.48 | 4.53 | 13 |
| GDA | 9615 | guanine deaminase | GDA | 2.05 | 3.70E-05 | 7.52 | 8.56 | 14 |
| PI3 | 5266 | peptidase inhibitor 3, skin-derived | PI3 | 2.03 | 2.50E-05 | 7.21 | 8.24 | 15 |
| SERPINB4 | 6318 | serpin peptidase inhibitor, clade B (ovalbumin), member 4 | SERPINB4 | 2 | 2.60E-05 | 4.46 | 5.46 | 16 |
| SERPINB3 | 6317 | serpin peptidase inhibitor, clade B (ovalbumin), member 3 | SERPINB3 | 1.98 | 0.0004 | 4.58 | 5.56 | 19 |
| SLC19A2 | 10560 | solute carrier family 19 (thiamine transporter), member 2 | SLC19A2 | 1.95 | 0.0009 | 8.2 | 9.17 | 21 |
| TFF1 | 7031 | trefoil factor 1 | TFF1 | 1.94 | 3.70E-05 | 10.99 | 11.95 | 23 |
| FGFBP1 | 9982 | fibroblast growth factor binding protein 1 | FGFBP1 | 1.94 | 0.0005 | 4.62 | 5.57 | 23 |
| SPRR2D | 6703 | small proline-rich protein 2D | SPRR2D | 1.93 | 0.005 | 4.08 | 5.03 | 27 |
| LINC00452 | 643365 | long intergenic non-protein coding RNA 452 | LINC00452 | 1.93 | 0.0029 | 10.86 | 11.81 | 27 |
| CST1 | 1469 | cystatin SN | CST1 | 1.92 | 0.0011 | 13.83 | 14.77 | 29 |
| CFHR4 | 10877 | complement factor H-related 4 | CFHR4 | 1.86 | 0.0032 | 4.91 | 5.81 | 33 |
| CEACAM5 | 1048 | carcinoembryonic antigen-related cell adhesion molecule 5 | CEACAM5 | 1.84 | 0.0023 | 14.4 | 15.28 | 34 |
| LCN2 | 3934 | lipocalin 2 | LCN2 | 1.84 | 0.0007 | 7.29 | 8.18 | 34 |
| CATSPERB | 79820 | catsper channel auxiliary subunit beta | CATSPERB | 1.84 | 0.0034 | 6.05 | 6.93 | 34 |
| IFITM2 | 10581 | interferon induced transmembrane protein 2 | IFITM2 | 1.83 | 0.005 | 13.58 | 14.45 | 37 |
| TRIM31 | 11074 | tripartite motif containing 31 | TRIM31 | 1.83 | 4.80E-05 | 8.35 | 9.22 | 37 |
| ASIC2 | 40 | acid-sensing (proton-gated) ion channel 2 | ASIC2 | 1.82 | 0.0002 | 5.63 | 6.49 | 39 |
| HSPG2 | 3339 | heparan sulfate proteoglycan 2 | HSPG2 | 1.82 | 0.0104 | 4.3 | 5.17 | 39 |
| UGT2B11 | 10720 | UDP glucuronosyltransferase 2 family, polypeptide B11 | UGT2B11 | 1.8 | 0.011 | 4.61 | 5.45 | 43 |
| PPL | 5493 | periplakin | PPL | 1.74 | 0.0005 | 4.31 | 5.1 | 51 |
| EXPH5 | 23086 | exophilin 5 | EXPH5 | 1.74 | 0.0035 | 5.59 | 6.39 | 51 |
| CCL16 | 6360 | chemokine (C-C motif) ligand 16 | CCL16 | 1.73 | 0.0125 | 4.78 | 5.57 | 53 |
| SLC25A44 | 9673 | solute carrier family 25, member 44 | SLC25A44 | 1.73 | 0.0033 | 9.84 | 10.63 | 53 |
| IFITM3 | 10410 | interferon induced transmembrane protein 3 | IFITM3 | 1.73 | 0.001 | 12.61 | 13.41 | 53 |
| IFITM1 | 8519 | interferon induced transmembrane protein 1 | IFITM1 | 1.72 | 0.0012 | 10.27 | 11.05 | 59 |
| RPS3A | 6189 | ribosomal protein S3A | RPS3A | 1.71 | 0.0058 | 3.86 | 4.63 | 62 |
| YIPF3 | 25844 | Yip1 domain family, member 3 | YIPF3 | 1.7 | 0.0044 | 11.69 | 12.46 | 65 |
| ADH1B | 125 | alcohol dehydrogenase 1B (class I), beta polypeptide | ADH1B | 1.69 | 0.0007 | 6.16 | 6.92 | 68 |
| WNT10B | 7480 | wingless-type MMTV integration site family, member 10B | WNT10B | 1.69 | 0.0066 | 4.36 | 5.12 | 68 |
| TMEM45B | 120224 | transmembrane protein 45B | TMEM45B | 1.69 | 0.0007 | 5.09 | 5.85 | 68 |
| AQP3 | 360 | aquaporin 3 (Gill blood group) | AQP3 | 1.68 | 0.0003 | 8.72 | 9.47 | 71 |
| IL2RA | 3559 | interleukin 2 receptor, alpha | IL2RA | 1.68 | 0.0293 | 3.17 | 3.92 | 71 |
| MYL9 | 10398 | myosin, light chain 9, regulatory | MYL9 | 1.66 | 0.0009 | 6.25 | 6.97 | 79 |
| FAM46A | 55603 | family with sequence similarity 46, member A | FAM46A | 1.66 | 0.009 | 7.75 | 8.48 | 79 |
| IQCH | 64799 | IQ motif containing H | IQCH | 1.66 | 0.0058 | 6.73 | 7.46 | 79 |
| OR51E2 | 81285 | olfactory receptor, family 51, subfamily E, member 2 | OR51E2 | 1.65 | 0.0002 | 10.66 | 11.39 | 85 |
| PIP | 5304 | prolactin-induced protein | PIP | 1.64 | 0.0002 | 5.11 | 5.82 | 89 |
| SLC7A2 | 6542 | solute carrier family 7 (cationic amino acid transporter, y+ system), member 2 | SLC7A2 | 1.64 | 0.0012 | 11.76 | 12.47 | 89 |
| MAST4 | 375449 | microtubule associated serine/threonine kinase family member 4 | MAST4 | 1.64 | 0.0003 | 8.71 | 9.43 | 89 |
| SIDT1 | 54847 | SID1 transmembrane family, member 1 | SIDT1 | 1.63 | 0.0075 | 5.4 | 6.11 | 93 |
| OR5M11 | 219487 | olfactory receptor, family 5, subfamily M, member 11 | OR5M11 | 1.63 | 0.019 | 3.62 | 4.33 | 93 |
| RBM44 | 375316 | RNA binding motif protein 44 | RBM44 | 1.63 | 0.0125 | 3.74 | 4.45 | 93 |
| SLC4A4 | 8671 | solute carrier family 4, sodium bicarbonate cotransporter, member 4 | SLC4A4 | 1.61 | 0.0004 | 8.94 | 9.63 | 101 |
| CACFD1 | 11094 | calcium channel flower domain containing 1 | CACFD1 | 1.61 | 0.0062 | 6.2 | 6.89 | 101 |
| ATRNL1 | 26033 | attractin-like 1 | ATRNL1 | 1.61 | 0.0044 | 4.53 | 5.22 | 101 |
| EHF | 26298 | ets homologous factor | EHF | 1.61 | 0.0001 | 10.64 | 11.33 | 101 |
| KY | 339855 | kyphoscoliosis peptidase | KY | 1.6 | 0.0002 | 4.8 | 5.47 | 109 |
| TCERG1 | 10915 | transcription elongation regulator 1 | TCERG1 | 1.59 | 0.0069 | 4.23 | 4.9 | 114 |
| CRLF2 | 64109 | cytokine receptor-like factor 2 | CRLF2 | 1.59 | 0.0029 | 7.32 | 7.99 | 114 |
| RAB39B | 116442 | RAB39B, member RAS oncogene family | RAB39B | 1.59 | 0.0034 | 3.24 | 3.91 | 114 |
| SUN3 | 256979 | Sad1 and UNC84 domain containing 3 | SUN3 | 1.59 | 0.0132 | 4.13 | 4.8 | 114 |
| KIAA1755 | 85449 | KIAA1755 | KIAA1755 | 1.58 | 0.0042 | 4.47 | 5.13 | 121 |
| OR2T29 | 343563 | olfactory receptor, family 2, subfamily T, member 29 | OR2T29 | 1.58 | 0.0117 | 3.87 | 4.53 | 121 |
| CA11 | 770 | carbonic anhydrase XI | CA11 | 1.57 | 0.0016 | 5.09 | 5.74 | 125 |
| CEACAM7 | 1087 | carcinoembryonic antigen-related cell adhesion molecule 7 | CEACAM7 | 1.57 | 0.0234 | 9.73 | 10.38 | 125 |
| DLG3 | 1741 | discs, large homolog 3 (Drosophila) | DLG3 | 1.57 | 0.0015 | 6.87 | 7.53 | 125 |
| EFHC2 | 80258 | EF-hand domain (C-terminal) containing 2 | EFHC2 | 1.57 | 0.0478 | 3.88 | 4.53 | 125 |
| PLEKHG4B | 153478 | pleckstrin homology domain containing, family G (with RhoGef domain) member 4B | PLEKHG4B | 1.57 | 0.0427 | 4.77 | 5.42 | 125 |
| RFX6 | 222546 | regulatory factor X, 6 | RFX6 | 1.57 | 0.0091 | 6.02 | 6.67 | 125 |
| TM4SF4 | 7104 | transmembrane 4 L six family member 4 | TM4SF4 | 1.56 | 0.0004 | 15.86 | 16.5 | 136 |
| TSHZ3 | 57616 | teashirt zinc finger homeobox 3 | TSHZ3 | 1.56 | 0.0227 | 4.01 | 4.65 | 136 |
| CCDC64 | 92558 | coiled-coil domain containing 64 | CCDC64 | 1.56 | 0.0232 | 6.17 | 6.81 | 136 |
| PLEKHG7 | 440107 | pleckstrin homology domain containing, family G (with RhoGef domain) member 7 | PLEKHG7 | 1.56 | 0.0015 | 2.85 | 3.49 | 136 |
| MAPK10 | 5602 | mitogen-activated protein kinase 10 | MAPK10 | 1.55 | 0.0035 | 4.27 | 4.9 | 147 |
| HEY1 | 23462 | hairy/enhancer-of-split related with YRPW motif 1 | HEY1 | 1.55 | 0.0004 | 5.79 | 6.42 | 147 |
| VPS37D | 155382 | vacuolar protein sorting 37 homolog D (S. cerevisiae) | VPS37D | 1.55 | 0.0265 | 7.03 | 7.66 | 147 |
| CKMT1A | 548596 | creatine kinase, mitochondrial 1A | CKMT1A | 1.55 | 0.0003 | 10.83 | 11.46 | 147 |
| CASP4 | 837 | caspase 4, apoptosis-related cysteine peptidase | CASP4 | 1.54 | 0.0003 | 8.79 | 9.41 | 159 |
| FFAR2 | 2867 | free fatty acid receptor 2 | FFAR2 | 1.54 | 0.0223 | 3.98 | 4.6 | 159 |
| GLRX2 | 51022 | glutaredoxin 2 | GLRX2 | 1.54 | 0.0098 | 9.06 | 9.68 | 159 |
| ARSJ | 79642 | arylsulfatase family, member J | ARSJ | 1.54 | 0.0005 | 6.86 | 7.49 | 159 |
| ASXL3 | 80816 | additional sex combs like 3 (Drosophila) | ASXL3 | 1.54 | 0.0028 | 3.5 | 4.13 | 159 |
| SESTD1 | 91404 | SEC14 and spectrin domains 1 | SESTD1 | 1.54 | 0.0034 | 8.48 | 9.1 | 159 |
| RABGGTA | 5875 | Rab geranylgeranyltransferase, alpha subunit | RABGGTA | 1.53 | 0.0268 | 10.11 | 10.72 | 171 |
| TCN1 | 6947 | transcobalamin I (vitamin B12 binding protein, R binder family) | TCN1 | 1.53 | 0.0237 | 7.4 | 8.01 | 171 |
| FAIM3 | 9214 | Fas apoptotic inhibitory molecule 3 | FCMR | 1.53 | 0.0022 | 3.75 | 4.36 | 171 |
| GGT6 | 124975 | gamma-glutamyltransferase 6 | GGT6 | 1.53 | 0.042 | 3.93 | 4.54 | 171 |
| CD300LD | 100131439 | CD300 molecule-like family member d | CD300LD | 1.53 | 0.0308 | 5.74 | 6.36 | 171 |
| IFI16 | 3428 | interferon, gamma-inducible protein 16 | IFI16 | 1.52 | 0.0042 | 5.37 | 5.98 | 184 |
| MMP1 | 4312 | matrix metallopeptidase 1 (interstitial collagenase) | MMP1 | 1.52 | 0.0238 | 5.25 | 5.85 | 184 |
| PRRG4 | 79056 | proline rich Gla (G-carboxyglutamic acid) 4 (transmembrane) | PRRG4 | 1.52 | 0.0083 | 6.27 | 6.87 | 184 |
| NEXN | 91624 | nexilin (F actin binding protein) | NEXN | 1.52 | 0.0287 | 4.49 | 5.1 | 184 |
| ATG4A | 115201 | autophagy related 4A, cysteine peptidase | ATG4A | 1.52 | 0.0011 | 7.32 | 7.92 | 184 |
| TMEM92 | 162461 | transmembrane protein 92 | TMEM92 | 1.52 | 0.0022 | 8.16 | 8.77 | 184 |
| AGT | 183 | angiotensinogen (serpin peptidase inhibitor, clade A, member 8) | AGT | 1.51 | 0.032 | 6.7 | 7.3 | 198 |
| ADAM12 | 8038 | ADAM metallopeptidase domain 12 | ADAM12 | 1.51 | 0.0017 | 7.99 | 8.59 | 198 |
| SLC17A4 | 10050 | solute carrier family 17 (sodium phosphate), member 4 | SLC17A4 | 1.51 | 0.0009 | 4.22 | 4.81 | 198 |
| CYP39A1 | 51302 | cytochrome P450, family 39, subfamily A, polypeptide 1 | CYP39A1 | 1.51 | 0.0092 | 3.96 | 4.56 | 198 |
| OSBPL5 | 114879 | oxysterol binding protein-like 5 | OSBPL5 | 1.51 | 0.0044 | 4.52 | 5.12 | 198 |
| TC2N | 123036 | tandem C2 domains, nuclear | TC2N | 1.51 | 0.004 | 12.89 | 13.48 | 198 |
| DLG4 | 1742 | discs, large homolog 4 (Drosophila) | DLG4 | 1.5 | 0.0418 | 6.34 | 6.93 | 208 |
| PKP4 | 8502 | plakophilin 4 | PKP4 | 1.5 | 0.0195 | 3.52 | 4.11 | 208 |
| TF | 7018 | transferrin | TF | -1.5 | 0.001 | 6.93 | 6.35 | 208 |
| IFT140 | 9742 | intraflagellar transport 140 homolog (Chlamydomonas) | IFT140 | -1.5 | 0.0474 | 7.56 | 6.97 | 208 |
| C1orf109 | 54955 | chromosome 1 open reading frame 109 | C1orf109 | -1.5 | 0.0195 | 8.63 | 8.04 | 208 |
| MAP3K5 | 4217 | mitogen-activated protein kinase kinase kinase 5 | MAP3K5 | -1.51 | 0.0079 | 7.93 | 7.34 | 198 |
| ZNF235 | 9310 | zinc finger protein 235 | ZNF235 | -1.51 | 0.0177 | 6.37 | 5.78 | 198 |
| ZNF331 | 55422 | zinc finger protein 331 | ZNF331 | -1.51 | 0.0029 | 7.8 | 7.2 | 198 |
| MYO19 | 80179 | myosin XIX | MYO19 | -1.51 | 0.0104 | 9.93 | 9.33 | 198 |
| CLCN2 | 1181 | chloride channel, voltage-sensitive 2 | CLCN2 | -1.52 | 0.0018 | 5.77 | 5.16 | 184 |
| MTAP | 4507 | methylthioadenosine phosphorylase | MTAP | -1.52 | 0.0411 | 7.05 | 6.45 | 184 |
| NDRG1 | 10397 | N-myc downstream regulated 1 | NDRG1 | -1.52 | 0.0073 | 8.92 | 8.32 | 184 |
| SERTAD4 | 56256 | SERTA domain containing 4 | SERTAD4 | -1.52 | 0.001 | 5.24 | 4.63 | 184 |
| CDT1 | 81620 | chromatin licensing and DNA replication factor 1 | CDT1 | -1.52 | 0.0025 | 7.29 | 6.68 | 184 |
| WDR73 | 84942 | WD repeat domain 73 | WDR73 | -1.52 | 0.0023 | 8.37 | 7.76 | 184 |
| TM4SF18 | 116441 | transmembrane 4 L six family member 18 | TM4SF18 | -1.52 | 0.0024 | 11.58 | 10.97 | 184 |
| C15orf40 | 123207 | chromosome 15 open reading frame 40 | C15orf40 | -1.52 | 0.001 | 7.72 | 7.12 | 184 |
| CKS1B | 1163 | CDC28 protein kinase regulatory subunit 1B | CKS1B | -1.53 | 0.0013 | 12.45 | 11.84 | 171 |
| GUCY1A2 | 2977 | guanylate cyclase 1, soluble, alpha 2 | GUCY1A2 | -1.53 | 0.0009 | 4.17 | 3.56 | 171 |
| SMO | 6608 | smoothened, frizzled family receptor | SMO | -1.53 | 0.0012 | 8.44 | 7.83 | 171 |
| SULT2B1 | 6820 | sulfotransferase family, cytosolic, 2B, member 1 | SULT2B1 | -1.53 | 0.0081 | 8.86 | 8.25 | 171 |
| ZNF175 | 7728 | zinc finger protein 175 | ZNF175 | -1.53 | 0.0483 | 8.44 | 7.82 | 171 |
| IGF2BP3 | 10643 | insulin-like growth factor 2 mRNA binding protein 3 | IGF2BP3 | -1.53 | 0.0013 | 11.2 | 10.58 | 171 |
| MORC1 | 27136 | MORC family CW-type zinc finger 1 | MORC1 | -1.53 | 0.0057 | 3.69 | 3.08 | 171 |
| CDPF1 | 150383 | cysteine-rich, DPF motif domain containing 1 | CDPF1 | -1.53 | 0.0089 | 9.21 | 8.6 | 171 |
| RAD9A | 5883 | RAD9 homolog A (S. pombe) | RAD9A | -1.54 | 0.0204 | 7.98 | 7.36 | 159 |
| DGKD | 8527 | diacylglycerol kinase, delta 130kDa | DGKD | -1.54 | 0.0008 | 7.4 | 6.78 | 159 |
| KNTC1 | 9735 | kinetochore associated 1 | KNTC1 | -1.54 | 0.0037 | 10.21 | 9.59 | 159 |
| VAV3 | 10451 | vav 3 guanine nucleotide exchange factor | VAV3 | -1.54 | 0.03 | 8.62 | 8 | 159 |
| PSAT1 | 29968 | phosphoserine aminotransferase 1 | PSAT1 | -1.54 | 0.0008 | 13.17 | 12.54 | 159 |
| DHRS1 | 115817 | dehydrogenase/reductase (SDR family) member 1 | DHRS1 | -1.54 | 0.0085 | 7.75 | 7.13 | 159 |
| ENO1 | 2023 | enolase 1, (alpha) | ENO1 | -1.55 | 0.0042 | 13.07 | 12.44 | 147 |
| HMBS | 3145 | hydroxymethylbilane synthase | HMBS | -1.55 | 0.0134 | 5.62 | 4.99 | 147 |
| ITGB6 | 3694 | integrin, beta 6 | ITGB6 | -1.55 | 0.0064 | 5.3 | 4.67 | 147 |
| RAF1 | 5894 | v-raf-1 murine leukemia viral oncogene homolog 1 | RAF1 | -1.55 | 0.0004 | 8.89 | 8.27 | 147 |
| GPC6 | 10082 | glypican 6 | GPC6 | -1.55 | 0.0159 | 5.51 | 4.88 | 147 |
| FBXL5 | 26234 | F-box and leucine-rich repeat protein 5 | FBXL5 | -1.55 | 0.0357 | 5.49 | 4.86 | 147 |
| TESC | 54997 | tescalcin | TESC | -1.55 | 0.0003 | 12.53 | 11.9 | 147 |
| CACNA2D4 | 93589 | calcium channel, voltage-dependent, alpha 2/delta subunit 4 | CACNA2D4 | -1.55 | 0.0424 | 4.05 | 3.41 | 147 |
| IVD | 3712 | isovaleryl-CoA dehydrogenase | IVD | -1.56 | 0.0175 | 7.64 | 7 | 136 |
| SLC3A1 | 6519 | solute carrier family 3 (cystine, dibasic and neutral amino acid transporters, activator of cystine, dibasic and neutral amino acid transport), member 1 | SLC3A1 | -1.56 | 0.0122 | 6.23 | 5.59 | 136 |
| SNAP25 | 6616 | synaptosomal-associated protein, 25kDa | SNAP25 | -1.56 | 0.0011 | 10.98 | 10.34 | 136 |
| MGEA5 | 10724 | meningioma expressed antigen 5 (hyaluronidase) | MGEA5 | -1.56 | 0.0022 | 3.55 | 2.91 | 136 |
| INTS1 | 26173 | integrator complex subunit 1 | INTS1 | -1.56 | 0.0079 | 9.41 | 8.77 | 136 |
| ALG6 | 29929 | asparagine-linked glycosylation 6, alpha-1,3-glucosyltransferase homolog (S. cerevisiae) | ALG6 | -1.56 | 0.0196 | 8.25 | 7.61 | 136 |
| ERAP1 | 51752 | endoplasmic reticulum aminopeptidase 1 | ERAP1 | -1.56 | 0.0067 | 4.33 | 3.69 | 136 |
| HIST1H1C | 3006 | histone cluster 1, H1c | HIST1H1C | -1.57 | 0.0096 | 10.26 | 9.6 | 125 |
| LIFR | 3977 | leukemia inhibitory factor receptor alpha | LIFR | -1.57 | 0.0157 | 7.69 | 7.04 | 125 |
| NAIP | 4671 | NLR family, apoptosis inhibitory protein | NAIP | -1.57 | 0.0178 | 6.01 | 5.36 | 125 |
| EBNA1BP2 | 10969 | EBNA1 binding protein 2 | EBNA1BP2 | -1.57 | 0.0176 | 4.7 | 4.05 | 125 |
| HKR1 | 284459 | HKR1, GLI-Kruppel zinc finger family member | HKR1 | -1.57 | 0.0018 | 5.56 | 4.91 | 125 |
| ADAM23 | 8745 | ADAM metallopeptidase domain 23 | ADAM23 | -1.58 | 0.0123 | 5.76 | 5.1 | 121 |
| LRRCC1 | 85444 | leucine rich repeat and coiled-coil centrosomal protein 1 | LRRCC1 | -1.58 | 0.0122 | 8.65 | 7.99 | 121 |
| PPM1B | 5495 | protein phosphatase, Mg2+/Mn2+ dependent, 1B | PPM1B | -1.59 | 0.0108 | 4.37 | 3.71 | 114 |
| LRIG1 | 26018 | leucine-rich repeats and immunoglobulin-like domains 1 | LRIG1 | -1.59 | 0.0004 | 4.85 | 4.18 | 114 |
| ANTXRL | 195977 | anthrax toxin receptor-like | ANTXRL | -1.59 | 0.0201 | 4.84 | 4.18 | 114 |
| BIRC3 | 330 | baculoviral IAP repeat containing 3 | BIRC3 | -1.6 | 0.0494 | 5.46 | 4.78 | 109 |
| DDIT4 | 54541 | DNA-damage-inducible transcript 4 | DDIT4 | -1.6 | 0.0097 | 12.64 | 11.96 | 109 |
| FLVCR2 | 55640 | feline leukemia virus subgroup C cellular receptor family, member 2 | FLVCR2 | -1.6 | 0.005 | 8.12 | 7.44 | 109 |
| TRPM6 | 140803 | transient receptor potential cation channel, subfamily M, member 6 | TRPM6 | -1.6 | 0.0429 | 5.73 | 5.05 | 109 |
| AXL | 558 | AXL receptor tyrosine kinase | AXL | -1.61 | 0.0464 | 12.09 | 11.4 | 101 |
| PHGDH | 26227 | phosphoglycerate dehydrogenase | PHGDH | -1.61 | 0.0005 | 8.55 | 7.86 | 101 |
| KLHL12 | 59349 | kelch-like 12 (Drosophila) | KLHL12 | -1.61 | 0.0036 | 9.44 | 8.75 | 101 |
| ZBTB45 | 84878 | zinc finger and BTB domain containing 45 | ZBTB45 | -1.61 | 0.0458 | 8.25 | 7.57 | 101 |
| HRH4 | 59340 | histamine receptor H4 | HRH4 | -1.62 | 0.0021 | 3.95 | 3.26 | 100 |
| MAP3K14 | 9020 | mitogen-activated protein kinase kinase kinase 14 | MAP3K14 | -1.63 | 0.0014 | 7.79 | 7.08 | 93 |
| KLF4 | 9314 | Kruppel-like factor 4 (gut) | KLF4 | -1.63 | 0.0299 | 5.37 | 4.67 | 93 |
| VWA1 | 64856 | von Willebrand factor A domain containing 1 | VWA1 | -1.63 | 0.0188 | 7.65 | 6.94 | 93 |
| MINA | 84864 | MYC induced nuclear antigen | MINA | -1.63 | 0.0023 | 10.03 | 9.32 | 93 |
| ANXA11 | 311 | annexin A11 | ANXA11 | -1.64 | 0.0001 | 14.44 | 13.73 | 89 |
| PCK2 | 5106 | phosphoenolpyruvate carboxykinase 2 (mitochondrial) | PCK2 | -1.65 | 0.0016 | 7.32 | 6.61 | 85 |
| SFRP1 | 6422 | secreted frizzled-related protein 1 | SFRP1 | -1.65 | 0.0006 | 6.85 | 6.13 | 85 |
| FAM208B | 54906 | family with sequence similarity 208, member B | FAM208B | -1.65 | 0.0143 | 9.18 | 8.46 | 85 |
| GALR1 | 2587 | galanin receptor 1 | GALR1 | -1.66 | 0.0046 | 5.08 | 4.35 | 79 |
| TIGD4 | 201798 | tigger transposable element derived 4 | TIGD4 | -1.66 | 0.0209 | 4.83 | 4.1 | 79 |
| GOLGA8O | 728047 | golgin A8 family, member O | GOLGA8O | -1.66 | 0.0028 | 6.22 | 5.49 | 79 |
| SERPINA6 | 866 | serpin peptidase inhibitor, clade A (alpha-1 antiproteinase, antitrypsin), member 6 | SERPINA6 | -1.67 | 0.0304 | 9.38 | 8.64 | 75 |
| PKP4 | 8502 | plakophilin 4 | PKP4 | -1.67 | 0.0037 | 5.62 | 4.88 | 75 |
| ERLIN2 | 11160 | ER lipid raft associated 2 | ERLIN2 | -1.67 | 0.0018 | 8.93 | 8.19 | 75 |
| FRMD6 | 122786 | FERM domain containing 6 | FRMD6 | -1.67 | 0.0434 | 5.86 | 5.12 | 75 |
| LPIN1 | 23175 | lipin 1 | LPIN1 | -1.68 | 0.0101 | 4.67 | 3.93 | 71 |
| OTUD3 | 23252 | OTU domain containing 3 | OTUD3 | -1.68 | 0.0123 | 7.01 | 6.26 | 71 |
| EFNA1 | 1942 | ephrin-A1 | EFNA1 | -1.7 | 0.0009 | 8.59 | 7.83 | 65 |
| OCRL | 4952 | oculocerebrorenal syndrome of Lowe | OCRL | -1.7 | 0.0199 | 4.38 | 3.61 | 65 |
| APOL2 | 23780 | apolipoprotein L, 2 | APOL2 | -1.71 | 0.0009 | 8.99 | 8.22 | 62 |
| ZNF419 | 79744 | zinc finger protein 419 | ZNF419 | -1.71 | 0.0023 | 6.56 | 5.78 | 62 |
| COL12A1 | 1303 | collagen, type XII, alpha 1 | COL12A1 | -1.72 | 0.0403 | 6.59 | 5.81 | 59 |
| GPRC5C | 55890 | G protein-coupled receptor, family C, group 5, member C | GPRC5C | -1.72 | 0.0024 | 6.56 | 5.78 | 59 |
| NAP1L1 | 4673 | nucleosome assembly protein 1-like 1 | NAP1L1 | -1.73 | 0.0005 | 5.94 | 5.14 | 53 |
| CIDEB | 27141 | cell death-inducing DFFA-like effector b | CIDEB | -1.73 | 0.0003 | 8.92 | 8.12 | 53 |
| LARP7 | 51574 | La ribonucleoprotein domain family, member 7 | LARP7 | -1.73 | 0.0202 | 3.69 | 2.89 | 53 |
| FBRS | 64319 | fibrosin | FBRS | -1.76 | 0.0025 | 9.1 | 8.28 | 50 |
| BUD31 | 8896 | BUD31 homolog (S. cerevisiae) | BUD31 | -1.78 | 0.0313 | 4.72 | 3.88 | 47 |
| PCDHB5 | 26167 | protocadherin beta 5 | PCDHB5 | -1.78 | 0.0077 | 5.07 | 4.23 | 47 |
| EGLN3 | 112399 | egl nine homolog 3 (C. elegans) | EGLN3 | -1.78 | 0.0014 | 8.98 | 8.14 | 47 |
| CA9 | 768 | carbonic anhydrase IX | CA9 | -1.79 | 0.0118 | 8.71 | 7.87 | 45 |
| CITED2 | 10370 | Cbp/p300-interacting transactivator, with Glu/Asp-rich carboxy-terminal domain, 2 | CITED2 | -1.79 | 0.0002 | 9.35 | 8.51 | 45 |
| TRIM9 | 114088 | tripartite motif containing 9 | TRIM9 | -1.8 | 0.0024 | 6.48 | 5.63 | 43 |
| TMEM141 | 85014 | transmembrane protein 141 | TMEM141 | -1.81 | 0.0004 | 13.27 | 12.42 | 42 |
| CCDC84 | 338657 | coiled-coil domain containing 84 | CCDC84 | -1.82 | 0.0002 | 6.64 | 5.77 | 39 |
| FAM13A | 10144 | family with sequence similarity 13, member A | FAM13A | -1.88 | 7.40E-05 | 8.87 | 7.95 | 32 |
| RAD54L | 8438 | RAD54-like (S. cerevisiae) | RAD54L | -1.9 | 0.0006 | 4.54 | 3.62 | 31 |
| ANKRD36 | 375248 | ankyrin repeat domain 36 | ANKRD36 | -1.91 | 0.0374 | 6.95 | 6.02 | 30 |
| BBS4 | 585 | Bardet-Biedl syndrome 4 | BBS4 | -1.94 | 0.0029 | 5.23 | 4.28 | 23 |
| LEPREL1 | 55214 | leprecan-like 1 | P3H2 | -1.94 | 1.70E-05 | 11.74 | 10.79 | 23 |
| TMEM2 | 23670 | transmembrane protein 2 | TMEM2 | -1.95 | 0.0001 | 7.09 | 6.13 | 21 |
| SKP2 | 6502 | S-phase kinase-associated protein 2, E3 ubiquitin protein ligase | SKP2 | -1.98 | 0.0007 | 5.33 | 4.34 | 19 |
| ADM | 133 | adrenomedullin | ADM | -1.99 | 0.0068 | 10.88 | 9.89 | 18 |
| MS4A18 | 728588 | membrane-spanning 4-domains, subfamily A, member 18 | MS4A18 | -2 | 0.0046 | 4.76 | 3.75 | 16 |
| TIAL1 | 7073 | TIA1 cytotoxic granule-associated RNA binding protein-like 1 | TIAL1 | -2.13 | 0.0002 | 6.39 | 5.3 | 9 |
| MAFK | 7975 | v-maf musculoaponeurotic fibrosarcoma oncogene homolog K (avian) | MAFK | -2.23 | 0.0001 | 9.21 | 8.05 | 6 |
| SLC2A14 | 144195 | solute carrier family 2 (facilitated glucose transporter), member 14 | SLC2A14 | -2.27 | 0.0043 | 13.07 | 11.88 | 4 |
| SLC2A3 | 6515 | solute carrier family 2 (facilitated glucose transporter), member 3 | SLC2A3 | -2.28 | 0.001 | 11.85 | 10.66 | 3 |
| FBXO17 | 115290 | F-box protein 17 | FBXO17 | -3.39 | 4.90E-07 | 10.14 | 8.38 | 1 |
