# Supplementary material for: FBXO17 promotes cell proliferation through activation of Akt in lung adenocarcinoma cells
Source: Respir Res. 2018 Oct 25;19:206. doi: 10.1186/s12931-018-0910-0 (PMC6203195; doi:10.1186/s12931-018-0910-0)
Supplement: Supplementary file 2 — Gene ontology enrichment analysis using ToppGene. (DOCX 21 kb) [file 12931_2018_910_MOESM2_ESM.docx]

**Table S2**

|  | | |  |  |  |  |  |  |  |  |  |  |
| --- | --- | --- | --- | --- | --- | --- | --- | --- | --- | --- | --- | --- |
| GO: Molecular function | | |  |  |  |  |  |  |  |  |  |  |
|  | ID | Name | pValue | FDR B&H | FDR B&Y | Bonferroni | Genes from Input | Genes in Annotation | |  |  |  |
| 1 | GO:0004866 | endopeptidase inhibitor activity | 1.92E-05 | 7.16E-03 | 5.12E-02 | 1.38E-02 | 10 | 171 |  |  |  |  |
| 2 | GO:0061135 | endopeptidase regulator activity | 2.58E-05 | 7.16E-03 | 5.12E-02 | 1.86E-02 | 10 | 177 |  |  |  |  |
| 3 | GO:0030414 | peptidase inhibitor activity | 2.98E-05 | 7.16E-03 | 5.12E-02 | 2.15E-02 | 10 | 180 |  |  |  |  |
| 4 | GO:0004869 | cysteine-type endopeptidase inhibitor activity | 4.00E-05 | 7.21E-03 | 5.16E-02 | 2.88E-02 | 6 | 58 |  |  |  |  |
| 5 | GO:0061134 | peptidase regulator activity | 1.71E-04 | 2.47E-02 | 1.77E-01 | 1.24E-01 | 10 | 222 |  |  |  |  |
|  |  |  |  |  |  |  |  |  |  |  |  |  |
|  | ID | Name | pValue | FDR B&H | FDR B&Y | Bonferroni | Genes from Input | Genes in Annotation | |  |  | |
| 1 | GO:0010951 | negative regulation of endopeptidase activity | 8.19E-08 | 2.70E-04 | 2.36E-03 | 2.89E-04 | 15 | 245 |  |  |  | |
| 2 | GO:0010466 | negative regulation of peptidase activity | 1.53E-07 | 2.70E-04 | 2.36E-03 | 5.40E-04 | 15 | 257 |  |  |  | |
| 3 | GO:0052548 | regulation of endopeptidase activity | 3.27E-07 | 3.84E-04 | 3.36E-03 | 1.15E-03 | 18 | 393 |  |  |  | |
| 4 | GO:0052547 | regulation of peptidase activity | 7.47E-07 | 6.59E-04 | 5.76E-03 | 2.64E-03 | 18 | 416 |  |  |  | |
| 5 | GO:0051346 | negative regulation of hydrolase activity | 3.43E-06 | 2.42E-03 | 2.12E-02 | 1.21E-02 | 17 | 417 |  |  |  | |
|  |  |  |  |  |  |  |  |  |  |  |  | |
|  |  |  |  |  |  |  |  |  |  |  |  | |
|  | ID | Name | pValue | FDR B&H | FDR B&Y | Bonferroni | Genes from Input | Genes in Annotation | |  |  | |
| 1 | 138045 | HIF-1-alpha transcription factor network | 2.12E-07 | 1.64E-04 | 1.19E-03 | 1.64E-04 | 7 | 65 |  |  |  | |
| 2 | 413350 | Serine biosynthesis, glycerate-3P => serine | 1.28E-04 | 4.95E-02 | 3.58E-01 | 9.91E-02 | 2 | 3 |  |  |  | |
|  |  |  |  |  |  |  |  |  |  |  |  | |
|  |  |  |  |  |  |  |  |  |  |  |  | |
|  |  |  |  |  |  |  |  |  |  |  |  | |
|  |  |  |  |  |  |  |  |  |  |  |  | |
|  |  |  |  |  |  |  |  |  |  |  |  | |
|  |  |  |  |  |  |  |  |  |  |  |  | |
|  |  |  |  |  |  |  |  |  |  |  |  | |
|  |  |  |  |  |  |  |  |  |  |  |  | |
|  |  |  |  |  |  |  |  |  |  |  |  | |
|  |  |  |  |  |  |  |  |  |  |  |  | |
|  |  |  |  |  |  |  |  |  |  |  |  | |
|  |  |  |  |  |  |  |  |  |  |  |  | |
|  |  |  |  |  |  |  |  |  |  |  |  | |
